# Development and Validation of a Smartphone Addiction Scale (SAS)

**DOI:** 10.1371/journal.pone.0056936

**Published:** 2013-02-27

**Authors:** Min Kwon, Joon-Yeop Lee, Wang-Youn Won, Jae-Woo Park, Jung-Ah Min, Changtae Hahn, Xinyu Gu, Ji-Hye Choi, Dai-Jin Kim

**Affiliations:** 1 Addiction Clinic, Department of Psychiatry, Seoul St. Mary's Hospital, The Catholic University of Korea, Seoul, South Korea; 2 College of Medicine, The Catholic University of Korea, Seoul, South Korea; 3 Department of Psychiatry, Seoul St. Paul's Hospital, College of Medicine, The Catholic University of Korea, Seoul, South Korea; 4 Department of Psychology, College of Social Science, The Catholic University of Korea, Bucheon, South Korea; 5 Department of Psychiatry, Seoul St. Mary's Hospital, College of Medicine, The Catholic University of Korea, Seoul, South Korea; 6 Addiction Research Institute, Department of Psychiatry, Seoul St. Mary's Hospital, The Catholic University of Korea, Seoul, South Korea; Federal University of Rio de Janeiro Brazil

## Abstract

**Objective:**

The aim of this study was to develop a self-diagnostic scale that could distinguish smartphone addicts based on the Korean self-diagnostic program for Internet addiction (K-scale) and the smartphone's own features. In addition, the reliability and validity of the smartphone addiction scale (SAS) was demonstrated.

**Methods:**

A total of 197 participants were selected from Nov. 2011 to Jan. 2012 to accomplish a set of questionnaires, including SAS, K-scale, modified Kimberly Young Internet addiction test (Y-scale), visual analogue scale (VAS), and substance dependence and abuse diagnosis of DSM-IV. There were 64 males and 133 females, with ages ranging from 18 to 53 years (M = 26.06; SD = 5.96). Factor analysis, internal-consistency test, t-test, ANOVA, and correlation analysis were conducted to verify the reliability and validity of SAS.

**Results:**

Based on the factor analysis results, the subscale “disturbance of reality testing” was removed, and six factors were left. The internal consistency and concurrent validity of SAS were verified (Cronbach's alpha = 0.967). SAS and its subscales were significantly correlated with K-scale and Y-scale. The VAS of each factor also showed a significant correlation with each subscale. In addition, differences were found in the job (p<0.05), education (p<0.05), and self-reported smartphone addiction scores (p<0.001) in SAS.

**Conclusions:**

This study developed the first scale of the smartphone addiction aspect of the diagnostic manual. This scale was proven to be relatively reliable and valid.

## Introduction

The recent development of Internet-based smart instruments has brought about a groundbreaking change in the society. In South Korea, according to the report of Korea Communications Commission, the smartphone users have been estimated to be over 20 million, and according to Statistics Korea, roughly over half of 40 million people (15-year-olds) carry smartphones, which means that smart instruments have spread considerably [1].

It is surprising that a tiny smartphone is built on a mobile computing platform with a more advanced computing ability and connectivity. Modern smartphone models serve to combine the functions of portable media players, low-end compact digital cameras, pocket video cameras, and GPS navigation units. Lately, smartphones typically have the functions of high-resolution touch screens, Web browsers that can access and properly display standard Webpages, and high-speed data access via Wi-Fi and mobile broadband. These advantages have brought enormous convenience to the modern society, but considering that smartphones are sharing most aspects of the Internet, the addiction to smartphones is highly likely to cause physical and psychosocial problems as well as Internet addiction [2]–[4].

Adverse results caused by the overuse of smartphones can be easily seen in today's society. For example, pedestrians viewing smartphone videos when crossing the street, without checking the traffic signal, are in danger of getting hit by cars; fumbling with one's smartphone while driving may cause car accidents; and elementary-school-aged children are highly likely to be addicted to smartphone games as well as to Internet video games [5]. In addition, students cannot concentrate in class, and the average cost of mobile-phone usage is increasing [1].

In a survey conducted by Stanford University in 2010, it was found that in 200 iPhone-using students, the Apple smartphone (a typical smartphone brand) can be rather addictive for both its recent adoptees and its long-time users, and many users relied on iPhone as a part of their lifestyle. All in all, 10% of the participants were fully addicted to their iPhone, 34% ranked themselves almost addicted to it, and 6% said they were not addicted to it at all. Further, 75% admitted to sleeping next to their iPhone, and 69% reported that they were more likely to forget their wallet than their iPhone. Although the admission of severe addiction was not very high among the side effects of iPhone addiction, with 41% saying it would be “a tragedy” to lose their iPhone and 22% saying that it is “dangerously alluring,” the rates still indicated the addictive power of smartphones [6].

Moreover, media reports suggest that people are becoming more attached to their smartphones, with ensuing social difficulties. A Website called “Crackberry.com” includes an online forum for “abusers” to admit their “addiction,” and a notice board for Blackberry “users and abusers,” where thousands of users discuss their addicted smartphone use. These self-report evidences indicate that a large number of users may be experiencing unwanted reliance on their smartphones [7].

“Addiction” is defined in the dictionary as: (1) a functional abnormality of the body caused by food or pharmaceutical toxins; (2) a pathologic condition that one cannot tolerate without the continuous administration of alcohol or drugs; and (3) the status of not being able to rationally judge or distinguish due to certain ideas or objects. “Addiction,” however, commonly handled by neuropsychiatric departments, is a phenomenon that manifests tolerance, withdrawal symptoms, and dependence, accompanied by social problems [8], [9]. The term was once limited to drugs or substances, but it is now also applied to gambling, Internet, gaming, mobile-phone usage, and other behavioral addictions [10].

The project “Development of a Korean Smartphone Addiction Proneness Scale” carried out by National Information Society Agency aimed to shed light on the concept and perception of smartphone addiction. In the project, each subject group was assessed, and the subjects were divided into the high-risk group, the low- to medium-risk group, and the general group. According to the results report, the smartphone addiction rates of the high-risk group and of the low- to medium-risk group were 2.2 and 9.3%, respectively, in adolescents, and 1.0 and 6.7%, respectively, in adults [11]. This indicates that the smartphone-related problems are too critical to be neglected. The project, however, provided only a simple assessment, differentiating between dependence and abuse, based on psychiatric diagnosis. As such, there is an urgent need to develop a diagnostic scale for smartphone addiction.

As it has been hypothesized that smartphone addiction has many aspects that are similar to those of Internet addiction, and as such, the Internet addiction criteria must be considered when developing smartphone addiction criteria. As Internet addiction has brought about a series of negative results due to the rapid development of the IT industry and of its increasing usage rate, it has become a popular issue, and many researches have been conducted on it. Kimberly S. Young was the first researcher to have established a basis for Internet addiction criteria, which has been widely quoted since then. In South Korea, a Korean self-diagnostic program for Internet addiction (K-scale) resulted from a joint research effort by Korea Agency for Digital Opportunities and Seoul National University [12]–[15]. K-scale was developed from Kimberly Young's scale of 20 questions into a 40-item scale.

Despite the importance of the smartphone-related problems, at the time of this study, there had been no studies focusing on this issue. Hence, the purpose of this study was to develop a self-diagnostic scale that could distinguish smartphone addicts. Based on K-scale and the smartphone's characteristics, the first smartphone addiction scale (SAS) was developed. Then, after revising the scale via factor analysis, the final SAS was completed.

## Materials and Methods

### Participants

For the study participants, a total of 214 adults were selected from two companies and two universities in Gyeonggi-do and Seoul in South Korea. Seventeen of them, however, failed to complete the questionnaires, and thus, only 197 of them were included in this study. Of these, 64 were male and 133 were female, with ages ranging from 18 to 53 years (M = 26.06; SD = 5.96). Each participant provided a written informed consent after receiving a full explanation of the study's purpose and procedure as approved by the Institutional Review Board of Seoul St. Mary's Hospital. The study participants' demographic features are shown in Table 1.

**Table 1:** Sociodemographic characteristics and SAS scores (N = 197).

Variables		N (%)	SAS Mean±SD	*p*
Sex	Male	64(32.5%)	104.52±39.69	.170
	Female	133(67.5%)	112.67±36.42	
Level of education	High school	57(28.9%)	120.93±34.30^a^	.013
	College	13(6.6%)	100.08±40.18	
	University	99(50.3%)	110.06±38.54	
	Master, Doctor	26(13.2%)	90.61±32.61^a^	
	No answer	2(1.0%)	111.50±55.86	
Job	Student	86(43.7%)	116.84±36.32^a^	.016
	Employee	49(24.9%)	114.84±39.03	
	Professional	52(26.4%)	95.77±36.81^a^	
	No job	5(2.5%)	94.00±26.05	
	Others	5(3.0%)	109.80±32.10	
Main use	Internet	34(17.3%)	104.24±32.95	.189
	SNS	42(21.4%)	118.67±38.71	
	Phone	103(52.6%)	106.59±40.04	
	Game, etc.	18(8.7%)	118.76±23.98	
Self- report of addiction	Strongly disagree	6(3.1%)	79.67±19.83	.000
	Disagree	29(14.7%)	92.17±32.84^a^	
	Unsure	67(34.0%)	103.06±35.71^b^	
	Agree	82(41.6%)	122.63±38.09^ab^	
	Strongly agree	13(6.6%)	120.15±31.55	
DSM-IV	Abuse	38(19.3%)	127.32±44.66	
	Dependence	19(9.6%)	136.00±31.04	

a, b: Scheffé test (the means with the same letter were significantly different)

### Measurement

#### 1) First SAS

The first SAS was a modified version of K-scale, a 40-item scale for juveniles concerning Internet addiction. The term “Internet” was changed to “smartphone.” From there, two items were removed, and one item was altered as it was inappropriate for adults. In addition, 10 questions were added, which described the mobile and ubiquitous features of smartphones. All the items were revised by six smartphone addiction field professionals (two psychiatrists, two clinical psychologists, and two counseling psychologists).

All the 48 items were divided into seven subscales: daily-life disturbance, disturbance of reality testing, positive anticipation, withdrawal, cyberspace-oriented relationship, overuse, and tolerance. Each item was assigned 1–6 points. The internal-consistency test result (Cronbach's alpha) was 0.97.

#### 2) VAS scores of seven features

To verify the concurrent validity of the seven subscales of the first SAS, the VAS scores of seven features were added in the questionnaire. This scale was added to determine the subjective thoughts of the participants regarding the seriousness of their addiction. Each feature was described above. Each item was assigned 1–10 points. The Visual Analogue Scale is shown in Figure S1.

#### 3) K-scale

K-scale was added to verify the concurrent validity of SAS. K-scale was developed through the collaborative effort of Korea Agency for Digital Opportunities and Seoul National University. It was revised from Kimberly Young's scale of 20 questions into a 40-item, 4-point scale [14]. In this study, the internal-consistency test result (Cronbach's alpha) of K-scale was 0.95.

#### 4) Y-scale

To verify the concurrent validity of the first SAS, Y-scale was added, consisting of 20 items and scored based on a 5-point scale measuring Internet addiction, which was demonstrated to have strong internal reliability [12], [13]. As with the first SAS, the term “Internet” in Y-scale was changed to “smartphone.” In this study, the internal-consistency test result (Cronbach's alpha) of Y-scale was 0.95.

#### 5) Substance dependence and abuse diagnosis of DSM-IV

This scale consists of 11 items and was added to assess if the participants conformed to the diagnosis of substance dependence and abuse of DSM-IV. Seven items were included in substance dependence, and four items in substance abuse [16].

### Statistical Analysis

#### 1) Composition of the subscales of SAS and internal consistency

The subscales of SAS were identified via factor analysis. Maximum likelihood factor analysis and direct oblimin rotation were used. To identify the parametric distribution, the ranges of skewness and kurtosis were calculated. The skewness range was -.253∼1.899, and the kurtosis range was -1.261∼3.351. Both of them were appropriate to the hypothesis. The direct oblimin was used because the subscales were considered to have relationships. The factor loads that were less than 0.4 were ignored. The internal consistency of the scale and subscales was measured.

#### 2) Concurrent validity of SAS

To determine if this scale really represents the severity of smartphone addiction, partial correlation analysis was conducted by controlling the level of education between the subscales for SAS, K-scale, and Y-scale.

#### 3) Concurrent validity of the subscales for SAS

Partial correlation analysis was conducted by controlling the level of education between the subscales for SAS, and the VAS scores of six features were measured to verify the concurrent validity of the subscales. The subscales were organized by summing up the items included as relevant factors through factor analysis.

## Results

### Factor Structure and Internal Consistency of SAS

The factor analysis results are shown in Table 2. As the outcome of the first factor analysis was not acceptable, the subscale “disturbance of reality testing” was removed because question no. 9 was the only question in this subscale, which was not included in any of the factors. Another 14 questions also failed to fit any of the factors. Thus, six factors were finally left in the second factor analysis. They explained 60.99% of the whole scale. The Cronbach's alpha for the total scale was 0.967, and those for the six factors were 0.858, 0.913, 0.876, 0.904, 0.825, and 0.865, respectively. The overall sampling adequacy of the 48-item scale was tested using Kaiser-Meyer-Olkin, and a high value of.936 was reported. The P-value of the Bartlett test was.000, which indicated that factor analysis was appropriate.

**Table 2:** Factor analysis of SAS.

No.	Question		Factor					
		M(SD)	Daily-life disturbance	Positive anticipation	Withdrawal	Cyberspace- oriented relationship	Overuse	Tolerance
1	Missing planned works due to smartphone usage	2.46(1.28)	-.406					
2	Having a hard time concentrating in class, while doing assignments, or while working due to smartphone use	2.98(1.34)	-.420					
3	Experiencing lightheadedness or blurred vision due to excessive smartphone use	2.96(1.44)	-.906					
4	Feeling pain in the wrists or at the back of the neck while using a smartphone	2.64(1.47)	-.788					
5	Feeling tired and lacking adequate sleep due to excessive smartphone use	2.63(1.43)	-.710					
6	Being incapable of doing anything without a smartphone as all schedules and personal stuff are saved in the smartphone	2.66(1.36)						
7	Neglecting matters other than smartphone use even when there are many other things to be done	2.41(1.29)						
8	Conflicting with family members due to smartphone use	1.61(0.95)						-.577
9	Experiencing auditory hallucinations of smartphone sounds while not using a smartphone	1.80(1.11)						
10	Feeling calm or cozy while using a smartphone	1.94(1.05)		.610				
11	Feeling pleasant or excited while using a smartphone	2.82(1.35)		.466				
12	Feeling confident while using a smartphone	2.13(1.20)		.622				
13	Being able to get rid of stress with smartphone use	2.17(1.15)		.634				
14	There is nothing other than smartphone use that is fun to do in my life.	1.73(0.96)		.625				
15	Having used a smartphone just to feel good	2.72(1.45)						
16	My life would be empty without my smartphone.	2.03(1.18)		.453				
17	Feeling most liberal while using a smartphone	1.96(1.16)		.612				
18	Smartphone use is the most fun thing to do.	1.70(0.96)		.687				
19	Won’t be able to stand not having a smartphone	2.18(1.30)			.622			
20	Feeling impatient and fretful when I am not holding my smartphone	2.13(1.25)			.641			
21	Having my smartphone in my mind even when I’m not using it	2.06(1.16)			.637			
22	I will never give up using my smartphone even when my daily life is already greatly affected by it.	2.05(1.16)			.449			
23	Getting irritated when bothered while using my smartphone	2.04(1.17)			.400			
24	Bringing my smartphone to the toilet even when I am in a hurry to get there	2.78(1.47)			.422			
25	Feeling depressed, anxious, or oversensitive when I am not able to use my smartphone	1.91(1.11)						
26	Being stressed out when I am not in a hot zone (Wi-Fi area)	2.47(1.41)						
27	Always preparing my charging pack to make sure that my smartphone is charged all the time	3.16(1.44)						
28	Feeling bored while doing other stuff without my smartphone	2.69(1.40)						
29	Feeling more relieved with my smartphone by my bedside when going to bed	2.50(1.29)						
30	Feeling great meeting more people via smartphone use	2.72(1.39)				.481		
31	Feeling that my relationships with my smartphone buddies are more intimate than my relationships with my real-life friends	1.77(1.03)				.658		
32	Not being able to use my smartphone would be as painful as losing a friend	1.92(1.13)				.703		
33	Feeling that my smartphone buddies understand me better than my real-life friends	1.54(0.86)				.725		
34	Constantly checking my smartphone so as not to miss conversations between other people on Twitter or Facebook	1.94(1.19)				.685		
35	Checking SNS (Social Networking Service) sites like Twitter or Facebook right after waking up	2.05(1.36)				.728		
36	Preferring to talk with my smartphone buddies to hanging out with my real-life friends or with the other members of my family	1.58(0.99)				.658		
37	Not minding spending money on paid smartphone applications	1.91(1.24)						
38	Trying to hide what I have been up to in relation to my smartphone	1.86(1.16)						
39	Not being able to keep appointments due to excessive smartphone use	1.61(0.92)						
40	Having used my smartphone when I am not supposed to (in class, during a meeting, etc.)	3.32(1.56)						
41	Preferring searching from my smartphone to asking other people	3.34(1.43)					.420	
42	My fully charged battery does not last for one whole day.	3.48(1.59)					.516	
43	Using my smartphone longer than I had intended	3.18(1.39)					.701	
44	Feeling the urge to use my smartphone again right after I stopped using it	2.59(1.31)					.469	
45	Having tried time and again to shorten my smartphone use time but failing all the time	1.94(1.07)						-.591
46	Always thinking that I should shorten my smartphone use time	2.16(1.31)						-.676
47	The people around me tell me that I use my smartphone too much.	1.70(1.03)						-.715
48	Preferring Web surfing on my smartphone to doing so on computers	2.05(1.41)						
Eigenvalue			20.021	2.871	1.985	1.656	1.429	1.312
Variance (%)			41.711	5.981	4.135	3.450	2.976	2.734

* A cutoff of 0.40 was used for inclusion.

### Concurrent Validity of SAS: Correlations between the Subscales of SAS and K-scale

The results of the partial correlation analysis that was conducted by controlling the level of education between the subscales of SAS and K-scale are shown in Table 3. The results show that all the subscales of SAS were significantly related to those of K-scale.

**Table 3:** Concurrent validity of SAS: Partial correlation analysis by controlling the level of education between the subscales of the SAS, K-scale, Y-scale, and VAS (N = 197).

Factor	K-scale	Y-scale	VAS
F1. Daily-life disturbance	.594**	.403**	.454**
F2. Positive anticipation	.536**	.461**	.361**
F3. Withdrawal	.607**	.495**	.568**
F4. Cyberspace-oriented relationship	.494**	.400**	.340**
F5. Overuse	.442**	.377**	.315**
F6. Tolerance	.582**	.347**	.357**

** p<.01

### Concurrent Validity of SAS: Correlations between the Subscales of SAS and Y-scale

The results of the partial correlation analysis that was conducted by controlling the level of education between the subscales of SAS and Y-scale are shown in Table 3. The results also show that all the subscales were positively related to those of Y-scale.

### Concurrent Validity of the SAS subscales

Partial correlation analysis was conducted by controlling the level of education of each subscale and VAS of six features. The results are shown in Table 3. Each couple was significantly related.

### SAS Scores of the Participants

As shown in Table 1, there was a significant difference between the high school level of education and the Master's or doctoral level of education (p = 0.013). A significant job difference between a student and a professional was also found (p = 0.016). It was interesting that in “self-reported smartphone addiction,” significant differences were found between “agree” and “disagree” and between “unsure” and “agree” (p<0.001).

Eleven items were also developed based on the substance dependence and abuse diagnosis of DSM-IV to test the characteristics of smartphone addiction. Like substance addiction, the concept of smartphone addiction as a kind of behavioral addiction had a number of criteria similar to those of dependence and abuse in DSM-IV. The results are presented in Table 1. There were 38 (19.3%) participants who conformed to the diagnosis of abuse in DSM-IV, and the mean SAS score was 127.80. Nineteen (9.6%) participants conformed to the diagnosis of dependence in DSM-IV, and the mean SAS score was 134.42.

## Discussion

A total of 197 participants were included in this study. No gender difference was found in the SAS scores. The mean SAS score was 104.5 for the male participants and 112.7 for the female participants, and 110.02 for both. Significant differences in the SAS scores occurred, however, between jobs, level of education, and self-reported smartphone addiction. In the level of education, the SAS scores of the participants with only a high school education (120.93±34.30) were significantly higher than those of the Master's and doctoral-degree participants (90.61+32.61). Also, for jobs, the students' SAS scores (116.84±36.32) were significantly higher than those of the professionals (95.77±36.81). In addition, the participants who answered “agree” (122.63±38.09) to their smartphone addiction obtained significantly higher SAS scores than the participants who answered “unsure” (103.06±35.71) and “disagree” (92.17±32.84). This indicates that people with a low level of education, and students, are more likely to become addicted to smartphone use, and the participants' self-report smartphone addiction was showing a similar tendency with the SAS scores. This may be because people with a low level of education may lack self-control, which has been suggested as a risk factor for game addiction [17].

Although the main use of the smartphone was reported to be as a “phone” (53.2%), the focus was placed on the smartphone characteristics of advanced computing ability and Internet connectivity, which make the smartphone more than a “phone” but a smart multi-medium. When the scale was being developed, it was focused on Internet use rather than on mobile-phone use, and on the analysis of the addiction concepts such as craving, tolerance, and withdrawal in the process of the concept analysis of smartphone addiction. Recently, in relation to the popular issue of smartphone addiction especially in South Korea, it has been investigated that the multi-functions of the smartphone, particularly Internet-based gaming and SNS problems, are becoming increasingly serious. Especially, the smartphone has the advantages of portability, real-time Internet searching, and convenient and interactive communication via SNS, in which differences from Internet addiction can be found.

The present study proposed an SAS. To identify the internal consistency of such SAS, it was administered to the 197 study subjects, and the Cronbach's alpha (0.967) was measured. Factor analysis was also performed, and the results are presented in Table 2. The SAS consisted of six factors, as follows: daily-life disturbance, positive anticipation, withdrawal, cyberspace-oriented relationship, overuse, and tolerance.

“Daily-life disturbance” includes missing planned work, having a hard time concentrating in class or while working, suffering from lightheadedness or blurred vision, pain on the wrists or at the back of the neck, and sleeping disturbance. It is understood that smartphones have already become a crucial part of the smartphone user's life. Smartphone users may experience difficulty in concentrating on their work because they cannot get their smartphone off their minds. Further, they spend so much time using their smartphone that they might already feel pain in their wrist, at the back of their neck, and in their eyes, head, etc. “Positive anticipation” is described as feeling excited about and getting rid of stress with smartphone use, and feeling empty without a smartphone. To most smartphone users, the smartphone is not just a calling device, game console, and PDA but also a friend because it brings them fun, relieves their exhaustion and anxieties, and makes them feel safe. “Withdrawal” involves being impatient, fretful, and intolerable without a smartphone, constantly having one's smartphone in one's mind even while not using it, never giving up using one's smartphone, and becoming irritated when bothered while using one's smartphone. “Cyberspace-oriented relationship” includes questions about the feeling that one's relationships with his/her friends obtained through a smartphone are more intimate than his/her relationships with his/her real-life friends, experiencing an uncontrolled feeling of loss when not able to use one's smartphone, and consequently constantly checking one's smartphone. For smartphone users, the smartphone world is a downsized real community or society formed by a Social Networking Service (SNS) site, such as Twitter or Facebook. “Overuse” refers to the uncontrollable use of one's smartphone, preferring to conduct searches using one's smartphone to asking help from other people, always preparing one's charging pack, and feeling the urge to use one's smartphone again right after one stopped using it. The last factor, “tolerance,” was defined as always trying to control one's smartphone use but always failing to do so. These six factors were consistent with the findings on the dimensions concerning the Internet addiction instruments, which are: “(1) compulsive Internet use and excessive time spent online (extent of compulsive Internet use and failure to control the amount of time spent on the Internet); (2) withdrawal symptoms (feelings of difficulty of coping, depression, or moodiness when being restricted from Internet use); (3) using the Internet for social comfort (using the Internet to seek social comfort and disposition towards using online social interaction to replace real-life interpersonal activities); and (4) negative consequences related to Internet use (negative outcomes such as social, academic, or work-related problems resulting from Internet use)” [13].

The concurrent validity was measured using partial correlation analysis, by controlling the level of education, to compare the SAS with Y-scale and K-scale. Each partial correlation coefficient was significantly high enough to prove the validity of the SAS. The internal consistency and concurrent validity of the scale and its subscales were also verified. The SAS and its subscales were significantly correlated with K-scale and Y-scale. The VAS score of each factor also showed a significant correlation with each subscale. All these indicate that the SAS was proven to represent the level of smartphone addiction with high reliability and validity.

The results of this study should be interpreted in the context of the study's limitations. First, the sample was small and was not randomized, and all the participants were adults and thus cannot represent the total population as high school students, for instance, are highly likely to have different features from adults. The further research should thus include adolescents. Second, the gender ratio was inappropriate. The number of women (133) was twice that of the men (64), and a gender difference error may exist. Thus, a well-designed research considering gender is necessary. Third, as the literature on this field is not yet rich enough, the theoretical base of this study was relatively weak. Fourth, because the characteristics of the mobile phone were overlooked in this study, in the further research, the validity problem in standardization should be confirmed by considering a comparison with mobile-phone addiction.

In spite of these limitations, the implications drawn from the results of this study expand the understanding of smartphone-related addictive behavior and provide a diagnostic manual for smartphone addiction. The SAS consists of 48 questions and is grouped into six subscales, all weighted equally on a 6-point scale. The six subscales' scores are summed up to yield a total SAS score with a 48–288 range, where a higher score indicates more serious smartphone addiction. Finally, the SAS was presented as including six factors and 33 items identified via factor analysis in Table S1.

Further studies should research on the correlation between smartphone addiction and anxiety, depression, loneliness, low self-esteem, impulse, and social maladjustment to verify the structural model of the diagnostic system of smartphone addiction. Moreover, it is necessary to develop a short form of SAS to enhance its reliability and convenience. In short, this research supplied the first SAS, which may serve as an opening for the clinical diagnosis of smartphone addiction.

## Supporting Information

Figure S1 Visual Analogue Scale.
(XLSX)

Table S1 English Version of SAS.
(XLSX)
